# The associations between lipid profiles and visceral obesity among gastrointestinal cancer patients: a cross-sectional study

**DOI:** 10.1186/s12944-022-01707-w

**Published:** 2022-10-14

**Authors:** Bo Gao, Xiangrui Li, Wenqing Chen, Shu’an Wang, Jian He, Yu Liu, Chao Ding, Xiaotian Chen

**Affiliations:** 1 Department of Clinical Nutrition, Nanjing Drum Tower Hospital, the Affiliated Hospital of Nanjing University Medical School, 321 Zhongshan Road, Gulou District, Nanjing, China; 2 Department of Radiology, Nanjing Drum Tower Hospital, the Affiliated Hospital of Nanjing University Medical School, Gulou District, 321 Zhongshan Road, Nanjing, China; 3 Department of Gynecology and Obstetrics, Women’s Hospital of Nanjing Medical University, Nanjing Maternity and Child Health Care Hospital, 1 Tianfei Road, Qinhuai District, Nanjing, China; 4 Department of General Surgery, Nanjing Drum Tower Hospital, the Affiliated Hospital of Nanjing University Medical School, Gulou District, 321 Zhongshan Road, Nanjing, China

**Keywords:** Gastrointestinal cancer, Visceral obesity, Lipid profiles, Diagnostic

## Abstract

**Background:**

Visceral obesity is associated with cancer incidence and prognosis. Altered lipid profiles are frequently seen in visceral obese patients. The blood test of lipid profiles is more convenient and has no radical side effects than computed tomography (CT), which is presently the most accurate way to measure visceral fat area. This article aims to investigate the associations between lipid profiles and visceral obesity in gastrointestinal cancer patients.

**Methods:**

In total, 399 patients newly diagnosed with gastrointestinal cancer were enrolled in this observational study. Lipid profiles were obtained from blood samples, and visceral fat mass area (VFA) was measured by CT. VFA ≥ 100 cm^2^ was considered visceral obesity. The area under the receiver operating characteristic curve (AUROC) was utilized to evaluate the prognostic powers of lipid parameters for viscerally obese gastrointestinal cancer patients.

**Results:**

Patients who had visceral obesity had higher triglyceride (TG) levels (1.20 ± 0.60 *vs.* 0.87 ± 0.57 mmo/L, *P* < 0.001), total cholesterol (TC) levels (3.57 ± 0.84 *vs.* 3.40 ± 0.82, *P* = 0.044), and low-density lipoprotein (LDL-C) levels (2.08 ± 0.66 *vs.* 1.94 ± 0.66, *P* = 0.047) and lower high-density lipoprotein (HDL-C) levels (0.88 ± 0.24 *vs.* 1.00 ± 0.26, *P* < 0.001) than those in the normal group. TG was positively correlated with VFA (r = 0.299, *P* < 0.001), while HDL-C was inversely correlated with VFA (r = -0.237, *P* < 0.001). TG and HDL-C had predictive capacity for visceral obesity at cutoff levels of 0.92 mmol/L (AUROC 0.700, 95% CI, 0.653–0.745, *P* < 0.001) and 0.98 mmol/L (AUROC 0.700, 95% CI, 0.585–0.682, *P* < 0.001), respectively. TG > 0.92 mmol/L with HDL-C < 0.98 mmol/L was linked with an increased risk of visceral obesity (OR = 4.068, 95% CI, 2.338—7.079, *P* < 0.001).

**Conclusions:**

Lipid profiles were significantly correlated with VFA. Gastrointestinal cancer patients with TG > 0.92 mmol/L and HDL-C < 0.98 mmol/L were at elevated risk of visceral obesity in the Chinese population. Identifying visceral obesity and taking proper actions in gastrointestinal cancers are helpful for overall tumor prognosis.

## Background

Cancer is a leading cause of death, and the incidence and mortality of cancer have rapidly increased in decades [1]. Gastrointestinal cancers are common types of malignant tumors that caused approximately 3.5 million newly diagnosed cases and 2.2 million new deaths worldwide in 2020 [1]. Obesity is well defined as a hazard element for cardiovascular and metabolic syndrome [2, 3]. Visceral obesity has also been proven to have an adverse influence on the occurrence and development of a variety of tumors in recent studies [4, 5]. Adipose tissue exerts endocrine effects by secreting proinflammatory cytokines [6], which have been demonstrated to be associated with insulin resistance, excessive inflammation and carcinogenic processes in various cancers [7]. More importantly, visceral obesity increases the difficulty of abdominal surgery and the risk of postoperative complications, such as wound infection, anastomotic leakage, and pneumonia [8, 9]. In addition, patients with visceral obesity undergoing laparoscopic rectal resection had an increased risk of switching to open surgery [10]. Overall, visceral obesity plays a vital role in detecting the outcomes of gastrointestinal cancer patients. Hence, capturing visceral obesity in this population is essential.

At present, visceral fat mass area (VFA) segmentation and measurement by computer tomography (CT) scan at umbilical slice is still the gold standard in evaluating visceral obesity [11]. However, drawbacks such as radiation exposure and high cost restrict the wide application of body composition analysis by CT scans. Visceral obesity is believed to be closely related to dyslipidemia [12]. A previous study revealed that excessive accumulation of adipose tissue results in elevated levels of very low-density lipoprotein integration and excretion and reduced clearance of triglyceride-rich lipoproteins, causing elevated low-density lipoprotein cholesterol (LDL-C). Concurrently, low levels of high-density lipoprotein cholesterol (HDL-C) occur [13]. Several studies have revealed correlations between dyslipidemia and obesity, and diabetes [14, 15]. However, such a correlation among gastrointestinal cancer patients and the diagnostic criteria by lipid profiles remain unknown. Exploring the associations between lipid profiles and visceral obesity may provide us with potential indicators for the simple identification of visceral obesity.

The main purpose of this study was to explore the relevance between VFA and lipid profiles. Furthermore, we identified cutoff values for diagnosing criteria of visceral obesity in gastrointestinal cancer patients among the Chinese population, which could provide additional prognostic information for surgical and survival outcomes.

## Methods

### Study subjects

**Fig. 1:**
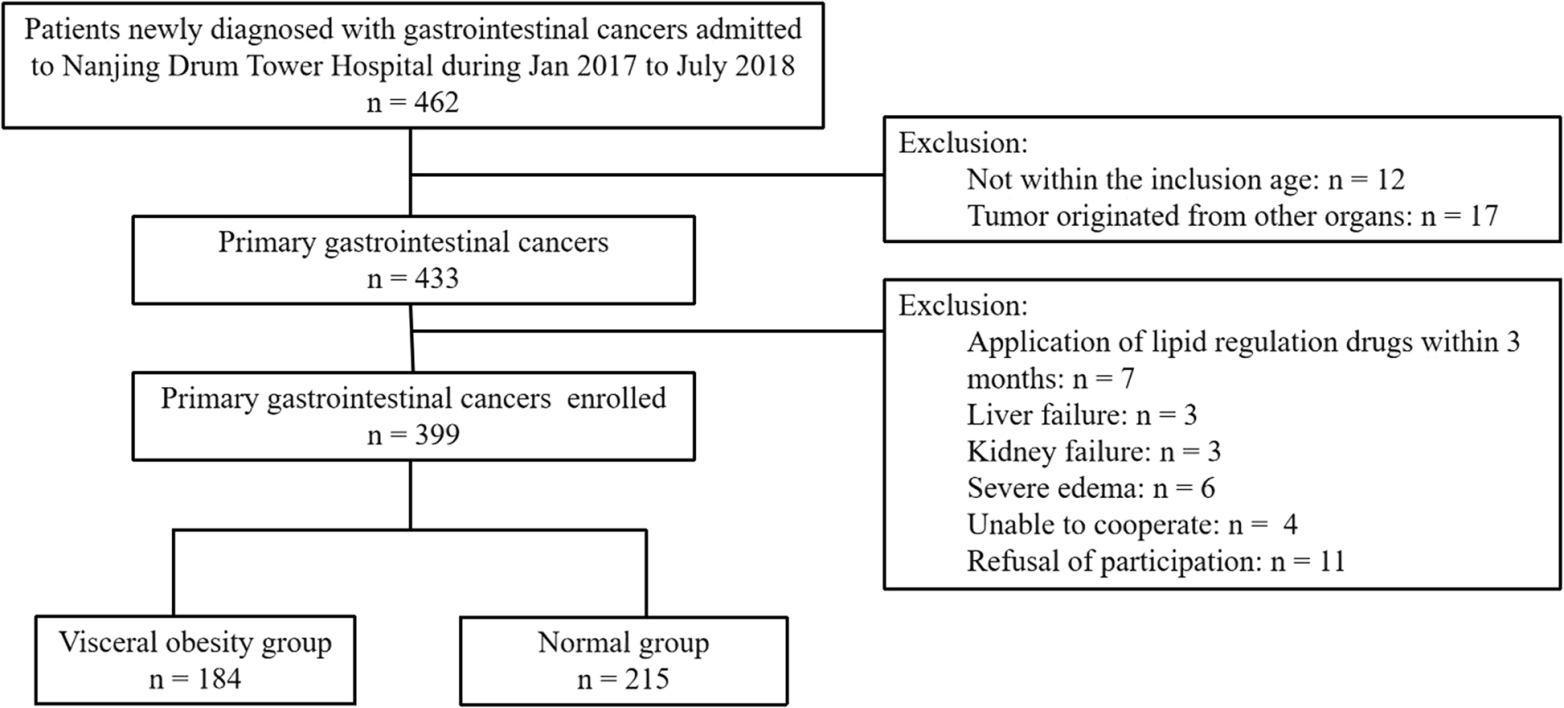
Flow chart of the study

A retrospective analysis was performed among patients aged from eighteen to eighty years who were newly diagnosed with gastrointestinal cancer. Patients who had primary tumors originating from other organs, received lipid regulation drugs within three months, had severe liver or renal dysfunction, had severe edema, or were unable or unwilling to undergo CT measurements were excluded from the study. From January 2017 to July 2018, a total of 399 subjects with complete data were finally included in this study. The study flowchart is displayed in Fig. 1. The cross-sectional study was ethically approved. Informed consent was exempted.

### Data collection

Fasting blood samples were taken at the morning of admission to determine the levels of lipids. Covariate parameters included white blood cell count, hemoglobin, albumin and tumor markers. The anthropometrics, previous history, health-related behaviors, tumor stage, tumor markers and nutritional conditions were assessed as well. Body mass index (BMI) was calculated as previously described [12]. Tumors were classified by the American Joint Committee on Cancer (AJCC) standard. Nutritional risk was recorded by the Nutritional Risk Score 2002 (NRS 2002). Nutritional severity was stratified by the Patient-Generated Subjective Global Assessment (PG-SGA). Nutritional scores, Eastern Cooperative Oncology Group Score (ECOG), and tumor characteristics were evaluated all by professionals.

### VFA measurement by CT

All included participants underwent abdominal CT scans at admission. According to a procedure reported previously, VFA was segmented and quantified at the level of umbilical slice level on the CT image [11]. VFA was manually outlined and calculated using MATLAB software (MathWorks, Massachusetts State, USA) independently by two professional radiologists. VFA was determined within the Hounsfield unit (HU) from -150 to -50. Based on the visceral obesity diagnostic standard by the Japan Society of Obesity [16], VFA exceeding 100 cm^2^ was defined as visceral obesity. Therefore, the participants were classified into two groups: the normal group and the visceral obesity group.

### Statistical analyses

For baseline characteristics, continuous parameters were expressed as the mean with standard deviation (SD). For categorical parameters, numbers and percentages were adopted. Continuous variables and categorical variables were analyzed to detect differences between groups using the independent Student test and χ2-test, respectively. The Pearson coefficient was used to identify the correlation of lipid profiles with VFA. A Bonferroni correction was applied to control type I error. Receiver operating characteristic (ROC) curves were utilized to appraise diagnostic performance and identify the cutoff values of lipid profiles. The DeLong test was adopted to compare the diagnostic powers between ROCs. The best cutoff values of lipid parameters were identified by the Youden index. The relationship between visceral obesity and associated lipid profiles was confirmed by regression analyses. *P* values below 0.05 were considered significantly different.

## Results

### Baseline

Finally, 399 patients were included. Two hundred fifteen (53.9%) patients were in the visceral obesity group, and 184 (46.1%) were in the normal group. The patient characteristics and demographics of the two groups are listed in Table 1. The normal group had significant differences from the visceral obesity group in terms of age and height. As expected, the BMI and weight of the normal group were significantly lower than those of the visceral obesity group (*P* < 0.001). With respect to tumor site, the distribution was significantly different (*P* < 0.05). The mean VFA in the two groups was 53.74 cm^2^ and 147.46 cm^2^, respectively (*P* < 0.001). Variables remained relatively balanced in other background factors.

**Table 1:** Baseline characteristics

	**Overall** ***N* = 399**	**Normal** ***N* = 215**	**Visceral obesity** ***N* = 184**	***P***
Age (year)	62.29 ± 11.95	61.00 ± 12.39	63.81 ± 11.26	0.019^**^
Male (n, %)	262 (65.7)	138 (64.2)	124 (67.4)	0.501
Height (cm)	164.07 ± 6.96	163.33 ± 6.36	164.94 ± 7.52	0.022^*^
Weight (kg)	63.78 ± 9.88	59.06 ± 8.30	69.32 ± 8.65	0.000^***^
BMI (kg/m^2^)	23.46 ± 3.70	22.00 ± 3.05	25.17 ± 3.67	0.000^***^
Diabetes (n, %)	26 (6.5)	14 (6.5)	12 (6.5)	0.997
Smoking history (n, %)	79 (19.8)	45 (20.9)	34 (18.5)	0.540
Drinking history (n, %)	41 (10.3)	23 (10.7)	18 (9.8)	0.764
Tumor location (n, %)				0.049^*^
Gastric cancer	290 (72.7)	165 (76.7)	125 (67.9)	
Colorectal cancer	109 (27.3)	50 (23.3)	59 (32.1)	
Tumor stage (AJCC) (n, %)				0.605
I	118 (29.6)	63 (29.3)	55 (29.9)	
II	139 (34.8)	71 (33.0)	68 (37.0)	
III	110 (27.6)	65 (30.2)	45 (24.5)	
IV	32 (8.0)	16 (7.4)	16 (8.7)	
ECOG	0.46 ± 0.58	0.47 ± 0.61	0.44 ± 0.54	0.608
NRS2002 ≥ 3	159 (39.8)	81 (37.7)	78 (42.4)	0.337
PG-SGA score	6.76 ± 3.52	6.91 ± 3.58	6.59 ± 3.45	0.366
Laboratory parameters				
White blood cells (× 10^9^/L)	5.81 ± 1.86	5.66 ± 1.87	5.99 ± 1.83	0.078
Hemoglobin (g/L)	124.11 ± 23.52	124.01 ± 22.64	124.24 ± 24.57	0.924
Albumin (g/L)	38.99 ± 4.15	38.94 ± 3.56	39.06 ± 4.75	0.769
CEA (ng/mL)	3.84 ± 8.73	4.00 ± 9.46	3.66 ± 7.81	0.703
CA125 (U/mL)	11.86 ± 21.34	12.92 ± 26.47	10.61 ± 12.84	0.294
CA199 (U/mL)	21.27 ± 42.90	19.34 ± 33.99	23.56 ± 51.50	0.357
CA724 (U/mL)	7.39 ± 22.98	5.84 ± 12.18	9.24 ± 31.27	0.181
CA242 (U/mL)	9.31 ± 15.14	8.27 ± 10.31	10.55 ± 19.35	0.167
Body composition				
Visceral fat mass area (cm^2^)	96.96 ± 57.53	53.74 ± 28.99	147.46 ± 38.16	0.000^***^
Visceral obesity (n, %)	184 (46.1)	–	–	

Abbreviations: *BMI* body mass index, *AJCC* American Joint Committee on Cancer, *ECOG* Eastern Cooperative Oncology Group Score, *NRS 2002* Nutritional Risk Score, *PG-SGA* Patient-Generated Subjective Global Assessment

^*^indicates statistically significant with *P* < 0.05; ^**^ indicates *P* value < 0.01; ***indicates *P* value < 0.001

### Comparison of lipid profiles between the visceral obesity and normal groups

All four lipid parameters showed significant divergences between groups (Table 2). Patients who had visceral obesity had higher TG levels (1.20 ± 0.60 *vs.* 0.87 ± 0.57 mmo/L, *P* < 0.001), TC levels (3.57 ± 0.84 *vs*. 3.40 ± 0.82, *P* = 0.044), LDL-C levels (2.08 ± 0.66 *vs*. 1.94 ± 0.66, *P* = 0.047) and lower HDL-C levels (0.88 ± 0.24 *vs*. 1.00 ± 0.26, *P* < 0.001) than those in the normal group.

**Table 2:** Comparison of lipid profiles between the visceral obesity group and the normal group among gastrointestinal cancer patients

	**Overall** ***N* = 399**	**Normal** ***N* = 215**	**Visceral obesity** ***N* = 184**	***P***
Triglyceride (mmol/L)	1.02 ± 0.61	0.87 ± 0.57	1.20 ± 0.60	0.000^***^
Cholesterol (mmol/L)	3.48 ± 0.83	3.40 ± 0.82	3.57 ± 0.84	0.044^*^
HDL-C (mmol/L)	0.95 ± 0.26	1.00 ± 0.26	0.88 ± 0.24	0.000^***^
LDL-C (mmol/L)	2.00 ± 0.66	1.94 ± 0.66	2.08 ± 0.66	0.047^*^

Abbreviations: *HDL-C* high-density lipoprotein cholesterol, *LDL-C* low-density lipoprotein cholesterol

^*^indicates statistically significant with *P* < 0.05; ^**^ indicates *P* value < 0.01; ***indicates *P* value < 0.001

### Correlation of lipid profiles with visceral obesity

As illustrated in Table 3, a positive association existed between VFA and TG levels (r = 0.299, *P* < 0.001) by Bonferroni’s correction in Pearson coefficient analysis. Conversely, VFA was strongly adversely associated with HDL-C levels (r = -0.237, *P* < 0.001) by Bonferroni’s correction in Pearson coefficient analysis.

**Table 3:** Correlation of lipid profiles with visceral fat mass area among gastrointestinal cancer patients

	**Pearson’s correlation**	**Visceral fat mass area**
Triglyceride	r	0.299
	*P*	0.000^***^
Cholesterol	r	0.105
	*P*	0.038
HDL-C	r	-0.237
	*P*	0.000^***^
LDL-C	r	0.112
	*P*	0.026

Abbreviations: *HDL-C* high-density lipoprotein cholesterol, *LDL-C* low-density lipoprotein cholesterol

^*^indicates statistically significant with *P* < 0.05; ^**^ indicates *P* value < 0.01; ***indicates *P* value < 0.001; Bonferroni’s correction: *p* < 0.0125

### The diagnostic values of lipid profile parameters for visceral obesity

**Fig. 2:**
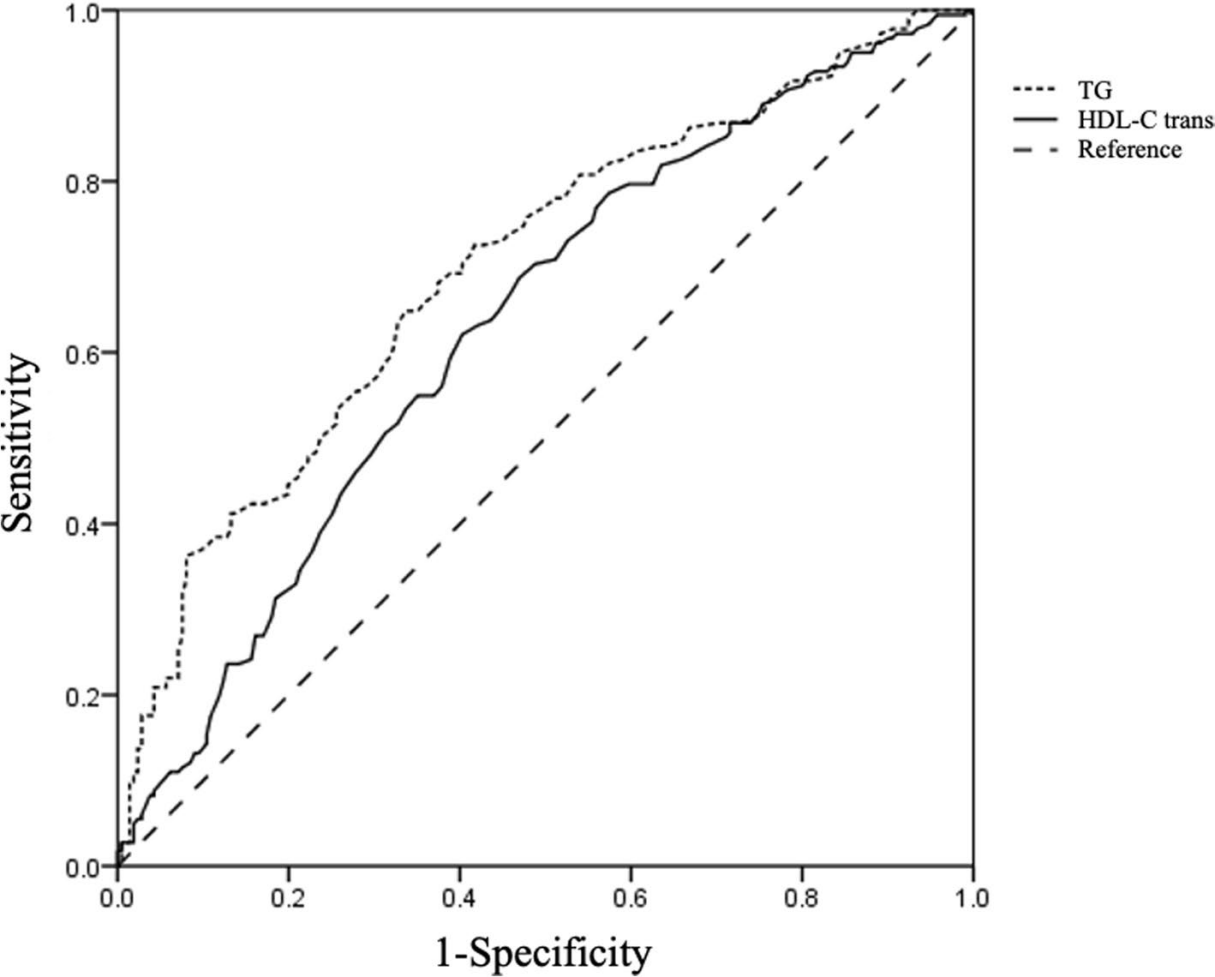
ROC analysis of TG and HDL-C to indicate visceral obesity among gastrointestinal cancer patients. For TG, the AUROC = 0.700 ± 0.03, 95% CI: 0.653–0.745, *P* < 0.001; cutoff value = 0.92, sensitivity = 65.22%, specificity = 66.98%. For HDL-C, the AUROC = 0.634 ± 0.03, 95% CI: 0.585–0.682, *P* < 0.001; cutoff value = 0.98, sensitivity = 69.02%, specificity = 53.95%. Abbreviations: *AUROC* area under receiver operating characteristic curve, *HDL-C* high-density lipoprotein cholesterol, *LDL-C* low-density lipoprotein cholesterol

As shown in Table 4 and Fig. 2, ROC analysis and AUROC measurements demonstrated that TG and HDL-C had significant and satisfactory diagnostic capacity for visceral obesity at levels of 0.92 mmol/L (AUROC 0.700; 95% CI, 0.653–0.745, *P* < 0.001) and 0.98 mmol/L (AUROC 0.634; 95% CI, 0.585–0.682, *P* < 0.001), respectively. The concentrations of triglyceride and HDL-C showed similar diagnostic capacity in capturing visceral obesity by the DeLong test (*P* = 0.061).

**Table 4:** ROCs of lipid profiles in diagnosing visceral obesity among gastrointestinal cancer patients

	**AUROC ± SE**	***P***	**95%CI**	**Cutoff value**	**Z**	***P* for Z**
Triglyceride (mmol/L)	0.700 ± 0.03	< 0.001^***^	0.653–0.745	0.92	–	–
Cholesterol (mmol/L)	0.554 ± 0.03	0.062	0.503–0.604	–	4.593	< 0.001^***^
HDL-C (mmol/L)	0.634 ± 0.03	< 0.001^***^	0.585–0.682	0.98	1.857	0.061
LDL-C (mmol/L)	0.554 ± 0.03	0.063	0.503–0.604	–	4.378	< 0.001^***^

Abbreviations: *AUROC* area under receiver operating characteristic curve, *HDL-C* high-density lipoprotein cholesterol, *LDL-C* low-density lipoprotein cholesterol

^*^indicates statistically significant with *P* < 0.05; ^**^ indicates *P* value < 0.01; ***indicates *P* value < 0.001; Z for DeLong test

### Logistic regression

Parameters that had significant differences in baseline characteristics were enrolled in logistic regression analysis (Table 5). The study discovered that age (OR = 1.033, 95% CI, 1.010–1.055, *P* = 0.004), BMI (OR = 1.442, 95% CI, 1.316–1.581, *P* < 0.001) and the combinations of TG > 0.92 mmol/L with HDL-C < 0.98 mmol/L (OR = 4.068, 95% CI, 2.338–7.079, *P* < 0.001) were independently associated with visceral obesity. The results indicated that patients who met the criteria of TG > 0.92 mmol/L and HDL-C < 0.98 mmol/L had a fourfold increased risk of visceral obesity.

**Table 5:** Univariate and multivariate logistic regression models for identifying visceral obesity among gastrointestinal cancer patients

	**Univariate regression**		**Multivariate regression**	
	OR (95%CI)	*P*	OR (95%CI)	*P*
Age	1.020 (1.003—1.038)	0.020^*^	1.033 (1.010—1.055)	0.004^**^
BMI	1.466 (1.339—1.606)	< 0.001^***^	1.442 (1.316—1.581)	< 0.001^***^
Tumor location	1.558 (1.001—2.425)	0.049^*^		
Triglyceride > 0.92 mmol/L with HDL-C < 0.98 mmol/L	4.177 (2.583—6.754)	< 0.001^***^	4.068 (2.338—7.079)	< 0.001^***^

Abbreviations: *HDL-C* high-density lipoprotein cholesterol

^*^indicates statistically significant with *P* < 0.05; ^**^ indicates *P* value < 0.01; ***indicates *P* value < 0.001

## Discussion

In this study, lipid profiles showed strong associations with VFA. Gastrointestinal cancer patients who are diagnosed with visceral obesity tend to have significantly higher triglyceride, cholesterol, and LDL-C levels and lower HDL-C concentrations. TG showed a significant positive correlation with VFA, while HDL-C showed a significant negative correlation with VFA in the present study. Both TG > 0.92 mmol/L and HDL-C < 0.98 mmol/L could predict visceral obesity in gastrointestinal cancers. Moreover, subjects with TG > 0.92 mmol/L and HDL-C < 0.98 mmol/L at the same time had a nearly fourfold risk for visceral obesity. The results of the present study indicated that TG and HDL-C may serve as potential simple diagnostic factors for visceral obesity among gastrointestinal patients in the Chinese population.

Obesity is associated with cancer incidence and outcomes [4, 5]. As an endocrine organ, adipose tissue secretes a large number of proinflammatory adipocytokines, such as interleukin-6 and tumor necrosis factor-alpha [6]. Obesity leads to an altered function of adipocytokines and further contributes to the progression of several malignancies [17]. Inflammation promotes tumor angiogenesis and accelerates metastasis via secretion and activation of the abovementioned adipocytokines [18]. Although both subcutaneous fat (ST) and visceral fat (VT) are correlated with cardiovascular risk and metabolism disorders, there are cellular and physiological differences between them [19, 20]. Compared with ST, VT is more insulin-resistant and has a greater capacity to predict mortality [19]. Visceral obesity was identified as a risk factor for several cancers, such as esophageal, pancreatic, and colorectal adenocarcinoma, associated with worse prognosis and higher recurrence and mortality rates [21, 22]. Recently, Park et al. evaluated 472 patients with stage III colorectal neoplasms by Cox regression and found that the VAT to total fat tissue ratio was strongly associated with cancer outcomes, indicating a fivefold risk of peritoneal seeding and tumor recurrence [23]. Keum et al. carried out a dose–response meta-analysis that enrolled 6 observational studies and 2776 subjects and found that every 25 cm^2^ increase in visceral adipose tissue area increased the odds of colorectal adenomas by 13% [24].

VFA is also associated with the surgical outcomes of gastrointestinal cancer patients [25]. Patients with obesity have a higher incidence of surgical and medical complications, such as mortality, infection, sepsis, and acute kidney injury [25, 26]. Chen et al. conducted a prospective study among 376 colorectal surgery subjects and discovered that age, excessive inner fat accumulation, and muscle attenuation were independent threats to postoperative complications [27]. In terms of gastrectomy, Yang et al. considered that patients with VFA equal to or greater than 100 cm^2^ encountered more intraoperative bleeding, prolonged operative time, and a higher occurrence rate of postoperative complications [28]. Increased abdominal fat tissue elevated the difficulties of gastrointestinal surgeries, since excessive visceral fat tissue may increase abdominal wall pressure, contribute to a normal anatomical field, and narrow the operative visibility, further resulting in a longer duration of operation and increased blood loss [25, 28]. Obese patients undergoing rectal resections are also at risk of anastomotic leakage and carry at least 1.2 times the odds of wound infection due to technical and metabolic factors [29, 30]. In detail, obesity patients had higher operative difficulties, while proinflammatory and insulin-resistance states may impair tissue repair and prolong wound healing, especially in the presence of malignancy [31]. Furthermore, visceral obesity patients often exhibit metabolic disorders. Metabolic disorders include insulin resistance and diabetes, lipid abnormalities, cardiovascular disease, and hypertension [6]. These comorbidities lead to increased health care difficulty, elevated mortality, and higher costs [32].

Since obesity affects cancer incidence and outcomes, assessing body fat, especially VAT, is overriding. Particularly for patients with operation indications, identifying patients with visceral obesity prior to surgery could facilitate the selection of an appropriate surgery type (laparoscopic surgery or open surgery) and provide intervention to reduce postoperative morbidities. Traditionally, anthropometric measurements such as BMI and waist circumference (WC) were easily performed to identify overweight and obese patients [33]. Nevertheless, BMI has some methodological bias and is obtained by the ratio of weight to height, which cannot distinguish fat mass from SAT and VAT [34]. WC and waist-to-hip ratio could also assess the degree of abdominal obesity [35] but remain with the same limitations. As reported, several methods can be used to detect human tissue distributions [36, 37]. CT is a common and noninvasive test method that accurately calculates the area of visceral and subcutaneous adipose tissue and is considered the most accurate way to measure abdominal fat compared with other methods [36, 37]. However, the high cost and ionizing radiation are not suitable for retesting CT in the short term. In addition, the accuracy of CT depends on the technique of the operator to a large extent. Additionally, CT is continuously having difficulties in arrangement and administration difficulty at hospitals and requires a long waiting time. Other body composition techniques have different drawbacks. Generally, MRI is more expensive than CT scans in China. The accuracy of BIA could be influenced by the individual’s hydration status, whereas pathological conditions of cancer could alter the hydration level [37].

In contrast, the blood test of lipid profiles was more convenient and had no radical side effects than CT measurement for oncology patients. Although CT is considered the gold standard for visceral obesity diagnosis, the blood test has superior repeatability and can be widely applied for frequent monitoring of visceral fat, which facilitates adjustment of treatment strategies. In addition, the frequency of blood sample collection is usually greater than that of CT tests along with the progression of the disease. According to the results of this study, lipid profiles could predict visceral fat, and the sensitivity and accuracy of TG and HDL were acceptable. A previous study was consistent with this result. Song et al. followed nondiabetic Japanese-Americans over 5 years and found that baseline plasma HDL-C concentration could independently predict the future accumulation of intra-abdominal fat [38]. Wadjchenberg's studies showed that visceral fat adipose tissue was related to blood lipids. The fluctuation trends of lipids with abdominal fat were consistent with the current research [39]. Elevated levels of TG and decreased levels of HDL are frequently seen in visceral obese patients. High plasma TG levels lead to an increased concentration of VLDL, which promotes the production of triglyceride-enriched HDL by cholesteryl ester transfer protein (CETP) [33]. Then, under the activity of hepatic triglyceride lipase, triglyceride-enriched HDL is degraded and removed from the blood [33]. This is the main reason for the low plasma HDL-C level found in patients with visceral obesity and hypertriglyceridemia.

In this study, age was also found to be significantly different between the two groups. Age, sex and inherited genes are predisposing factors for variation in VAT accumulation [33, 40]. Other than the above nonmodifiable factors, physical activity and dietary patterns are modifiable and provide economically, noninvasive and nonpharmacological approaches for VAT reduction and body composition improvement [41]. Hence, following visceral identification, immediate lifestyle intervention is beneficial to these cancer patients in terms of both surgical outcomes and cancer prognosis.

### Comparisons with other studies and what does the current work add to the existing knowledge

Former scientific issues were exploring the mechanism underlying lipid profiles and fat tissue [12]. Several studies have focused on the relationship between obesity and the prognosis of malignancies [8–10]. However, as a simple tool, the diagnostic value of lipid profiles in detecting visceral obesity was not considered. This study demonstrated that HDL-C and triglyceride levels could be used as simple and easily accessible indicators to recognize visceral obesity in gastrointestinal cancer. As previously reported [9], CT served as an accurate standard for evaluation of visceral fat area. However, the reality was that in most backward areas, professional radiologists who mastered body composition measurement by CT were in serious shortage. Consequently, a significant portion of cancer patients with visceral obesity might be neglected. Therefore, it is essential to find a simple tool for diagnosing visceral obesity, which is the significance of the current research. This should be the first study identifying the diagnostic point of lipids in diagnosing visceral obesity, which could greatly benefit cancer patients in preoperative evaluation and individualized nutritional support.

### Study strengths and limitations

There were several strengths in the present research. First, the sample size of the study design was sufficient. Second, the visceral fat area measurement and the visceral obesity criteria for comparison were based on CT scans, which are accurate for body composition evaluation. Third, the current study not only analyzed the correlations between lipids and inner fat but also identified the diagnostic point for diagnosis.

There were some limitations. First, this study was a single-center, cross-sectional design and was performed only among Chinese individuals. Second, the study adopted Japanese criteria of visceral obesity, which would limit the generalization of the conclusions worldwide. Third, the research was based on a retrospective study and might have certain biases.

## Conclusions

In conclusion, lipid profiles were significantly correlated with VFA. TG > 0.92 mmol/L combined with HDL-C < 0.98 mmol/L could diagnose visceral obesity in gastrointestinal cancers. Although CT scan measurement of VAT was accurate, its side effects and sophisticated segmentation would greatly restrict its wide application, especially in routine follow-up periods and nutritional consulting visits. Lipid profiles are useful and convenient biomarkers without radical side effects. The nutritional support strategies were different between patients with or without visceral obesity. Early determination of visceral obesity would be helpful for professionals to plan operative strategies, guide individuals with healthy living and eating habits in routine follow-up, and further promote patients’ overall tumor prognosis.

## Data Availability

The datasets analyzed in the present study are available from the corresponding authors on reasonable request.

## Abbreviations

CT: Computer tomography
VFA: Visceral fat mass area
AUROC: Area under receiver operating characteristic curves
TG: Triglyceride
TC: Total cholesterol
LDL-C: Low-density lipoprotein cholesterol
HDL-C: High-density lipoprotein cholesterol
VLDL: Very low-density lipoprotein
CEA: Carcinoembryonic antigen
CA125: Carbohydrate antigen 125
CA199: Carbohydrate antigen 199
CA724: Carbohydrate antigen 724
CA242: Carbohydrate antigen 242
BMI: Body mass index
ECOG: Eastern Cooperative Oncology Group Score
AJCC: American Joint Committee on Cancer
NRS 2002: Nutritional Risk Score 2002
PG-SGA: Patient-Generated Subjective Global Assessment
HU: Hounsfield unit
SD: Standard deviation
ROC: Receiver operating characteristic
AUROC: Area under receiver operating characteristic curves
OR: Odds ratio
Cl: Confidence intervals
SAT: Subcutaneous adipose tissue
VAT: Visceral adipose tissue
WC: Waist circumference
BIA: Bioelectrical impedance analysis
DXA: Dual-energy X-ray absorptiometry
CETP: Cholesteryl ester transfer protein
